# Stretching Reduces Skin Thickness and Improves Subcutaneous Tissue Mobility in a Murine Model of Systemic Sclerosis

**DOI:** 10.3389/fimmu.2017.00124

**Published:** 2017-02-16

**Authors:** Ying Xiong, Lisbeth Berrueta, Katia Urso, Sara Olenich, Igla Muskaj, Gary J. Badger, Antonios Aliprantis, Robert Lafyatis, Helene M. Langevin

**Affiliations:** ^1^Division of Preventive Medicine, Brigham and Women’s Hospital, Harvard Medical School, Boston, MA, USA; ^2^Division of Rheumatology, Immunology and Allergy, Brigham and Women’s Hospital, Harvard Medical School, Boston, MA, USA; ^3^Department of Medical Biostatistics, University of Vermont, Burlington, VT, USA; ^4^University of Pittsburgh, School of Medicine, Pittsburgh, PA, USA; ^5^Department of Neurological Sciences, University of Vermont, Burlington, VT, USA

**Keywords:** scleroderma, systemic sclerosis, GvHD, stretching, physical therapy, inflammation, fibrosis

## Abstract

**Objective:**

Although physical therapy can help preserve mobility in patients with systemic sclerosis (SSc), stretching has not been used systematically as a treatment to prevent or reverse the disease process. We previously showed in rodent models that stretching promotes the resolution of connective tissue inflammation and reduces new collagen formation after injury. Here, we tested the hypothesis that stretching would impact scleroderma development using a mouse sclerodermatous graft-versus-host disease (sclGvHD) model.

**Methods:**

The model consists in the adoptive transfer (allogeneic) of splenocytes from B10.D2 mice (graft) into Rag2^−/−^ BALB/c hosts (sclGvHD), resulting in skin inflammation followed by fibrosis over 4 weeks. SclGvHD mice and controls were randomized to stretching *in vivo* for 10 min daily versus no stretching.

**Results:**

Weekly ultrasound measurements of skin thickness and subcutaneous tissue mobility in the back (relative tissue displacement during passive trunk motion) successfully captured the different phases of the sclGvHD model. Stretching reduced skin thickness and increased subcutaneous tissue mobility compared to no stretching at week 3. Stretching also reduced the expression of CCL2 and ADAM8 in the skin at week 4, which are two genes known to be upregulated in both murine sclGvHD and the inflammatory subset of human SSc. However, there was no evidence that stretching attenuated inflammation at week 2.

**Conclusion:**

Daily stretching for 10 min can improve skin thickness and mobility in the absence of any other treatment in the sclGvHD murine model. These pre-clinical results suggest that a systematic investigation of stretching as a therapeutic modality is warranted in patients with SSc.

## Introduction

Systemic sclerosis (SSc, also known as scleroderma) is an autoimmune disorder characterized by chronic dysregulation of innate and adaptive immune systems, vasculopathy, and fibroblast dysfunction resulting in fibrosis (1). Although clinical manifestations of SSc are heterogeneous, their hallmark is skin fibrosis, including the dermis and subcutaneous tissue (1–3). When subcutaneous tissue becomes fibrotic, adhesions can form between the skin and underlying connective tissues, which leads to impaired movement between these tissue layers and decreased range of motion (4). Non-pharmacological treatments, including physical therapy and stretching, are thought to be important adjuncts to pharmacological treatment in patients with SSc (5). However, there is evidence that these types of interventions are underutilized, and stretching has not been used systematically to prevent or reverse the diseases process (6, 7). Conversely, the possibility that lack of movement may itself be an important contributor to the pathophysiology of the disease has not been investigated. Thus, the lack of a standardized approach to stretching, including the correct “dose,” and dearth of insight into the mechanisms engaged at the tissue level by stretching has limited the application of this potentially powerful, yet non-invasive treatment (7–9).

We have previously developed an animal model in which mice or rats spontaneously stretch their whole body when they are partially lifted by the tail and allowed to grasp the edge of a surface with their front paws (10). When held in this position, the animals spontaneously extend both front and hind limbs, which increases the distance between shoulders and hips by ~25%. Using this model, we showed that stretching promotes the resolution of inflammation in subcutaneous connective tissues of the back and decreases newly formed collagen in a subcutaneous connective tissue injury model (10–12).

Because inflammation and fibrosis are known to contribute to the development of SSc pathology, the goal of this study was to test the effect of daily stretching in a murine model of SSc. The mouse sclerodermatous Graft-versus-host disease (sclGvHD) has been demonstrated to mimic a subset of SSc patients with an inflammatory gene signature (13, 14). In this model Rag2^−/−^ BALB/c hosts (sclGvHD mice) receive adoptively transferred splenocytes from MHC-matched allogeneic B10.D2 mice (graft). The graft-versus-host reaction culminates in skin inflammation and fibrosis. Seven days after splenocyte transfer, the sclGvHD mice lose weight and inflammatory cells begin to infiltrate the skin. Over the course of the following 4 weeks, collagen accumulates in the skin and fibrosis is clinically manifested as alopecia (15) (15–17). We used novel high-frequency ultrasound methods to follow the course of the disease and measure the impact of stretching. Our ultrasound measures included the thickness of skin and subcutaneous tissue, as well as the relative motion between the skin and underlying subcutaneous connective tissue layers in dynamic ultrasound recordings during passive flexion of the trunk. We chose to focus our measurements on the skin and subcutaneous tissues of the back, which provide accessible flat tissue planes that can be measured reliably with both static and dynamic imaging as previously demonstrated in humans and large animals (18–20), and used high-frequency (50 MHz) ultrasound, which provides the high resolution needed to apply these techniques to rodents. We hypothesized that, in the absence of stretch, ultrasound measurement of skin thickness are increased and subcutaneous tissue mobility are decreased in sclGvHD compared with control mice. We further hypothesized that stretching attenuates these abnormalities in the sclGvHD mice. In addition, because sclGvHD demonstrates a gene expression pattern similar to the inflammatory subset of scleroderma (13, 14) and increased expression of extracellular matrix-associated pathways is evident in this inflammatory subset (13), we also examined the expressions of TGF-β, TIMP1, MMP-12, ADAM8, IL4RA, and CCL2 genes which have been previously shown to be upregulated in both murine sclGvHD and the inflammatory subset of SSc patients (13, 14, 21, 22).

## Materials and Methods

### Mice and sclGvHD Model

The animal testing protocol used in this study was approved by the Harvard Medical School Institutional Animal Care and Use Committee. *Rag2^−/−^* mice on a BALB/c genetic background were generated as described previously in Ref. (13, 23). BALB/c and B10.D2, mice were obtained from the Jackson Laboratory (Bar Harbor, ME, USA). All mice were housed in a specific pathogen-free animal facility at the Harvard School of Public Health. Mice were housed and maintained in accordance with the Guide for Care and Use of Laboratory Animals. The drinking water of all *Rag2^−/−^* mice was supplemented with sulfamethoxazole and trimethoprim (Sulfatrim, Hi-Tech Pharmacal, 0.6 mg/ml drinking solution). The sclGvHD model was established as described previously (15).

Six- to nine-week-old males were used for all the experiments. Briefly, 20–40 million BALB/c (syngeneic) or B10.D2 (allogeneic) red-blood-cell-free splenocytes were transferred *via* tail-vein injection into host mice. Injection of allogeneic splenocytes produced the sclGvHD phenotype, while injection of syngeneic splenocytes served as controls. The mice were weighed and scored clinically once a week by a blinded observer as follows: 0 = no evidence of disease, 1 = fur ruffling or hunched posture, 2 = alopecia < 25% of body surface area, 3 = alopecia > 25% of body surface area, and 4 = death or a veterinary order to euthanize. Half a point was added for periorbital swelling.

### Study Design

Mice were randomized into one of four groups (*N* = 12/group): syngeneic control/no stretch (Ctl-NS), syngeneic control/stretch (Ctl-S), sclGvHD/no stretch (Scl-NS), and sclGvHD/stretch (Scl-S). Allogeneic (sclGvHD) or Syngeneic (Control) splenocytes were injected on day 0. The control group was included to document the effect of scleroderma using ultrasound. The stretch (or no stretch) interventions were performed for 10 min once a day, 5 days per week, for 4 weeks, beginning 3 days after splenocyte injection. Ultrasound measurements were performed once a week during the 4-week intervention, except at week 0 due to barrier facility constraints. At all time points, ultrasound measurements were taken 1 h after the last stretching (or no stretching) session. At the end of week 4, mice were euthanized by decapitation under deep isoflurane anesthesia immediately after the last ultrasound measurements, and skin samples from the back were excised for histology and gene expression analysis after shaving the back.

### Intervention Methods

**Figure 1:**
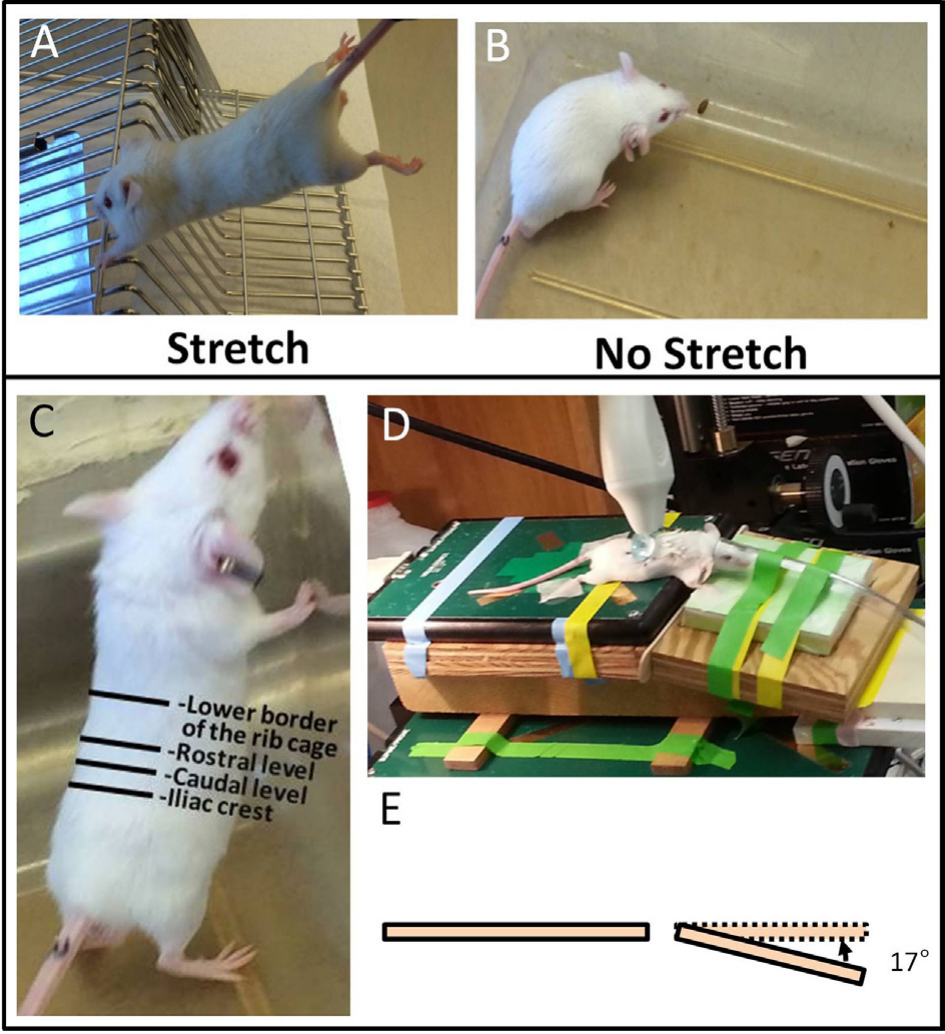
**Ultrasound imaging acquisition and stretching methods**. **(A,B)**: Stretching **(A)** and no stretching **(B)** interventions. **(C)**: Location of rostral and caudal levels for acquisition of transverse ultrasound images. **(D)**: Position of mouse on articulated table during acquisition of dynamic ultrasound imaging during upward and downward table motion. **(E)**: Angle of table motion.

*Stretching method*: mice were stretched by gently lifting them by the base of the tail until reaching ~45° angle to the horizontal. While grasping onto a bar with their front paws, the mice spontaneously extend their hind limbs. The stretching, which exerted traction on the whole back, increases the distance between the shoulder and hip by ~25% (10). With minimal habituating, mice were able to hold this position comfortably for 10 min without struggling, vocalizing, or other signs of distress (Figure 1A). *No stretching (sham)*: mice were removed from their cage for 10 min but were neither lifted nor stretched (Figure 1B).

### Ultrasound Data Acquisition

All ultrasound data acquisition and measurements were performed by investigators blinded to intervention condition. Ultrasound images of the back were acquired under isoflurane anesthesia. A high-frequency ultrasound scanner (Vevo 2100, Fujifilm VisualSonic, Toronto, ON, Canada) in B mode with a 50-MHz transducer (MS 700) was used for optimal spatial resolution, which provided a resolution of 30 μm × 75 μm (axial × lateral) and a focal length of 5.0 mm. A conductive gel was centrifuged for 5 min to remove air bubbles and spread over the skin. To ensure complete contact between the skin and the transducer, the fur in the back of the mice was parted after applying the gel and prior to imaging. This was done rather than shaving to avoid injury or irritation to the skin. The transducer was stabilized with a clamp and mounted into an articulated arm to control the distance and the angle between the transducer and the skin surface. For skin thickness measurements, the transducer was oriented transversely, perpendicular to the skin of the back and centered on the midline. Ultrasound images were acquired at two levels, one rostral and one caudal, on the left side of the back as shown in Figure 1C. For cumulative displacement measurement, the transducer was oriented longitudinally, 0.5 cm lateral and parallel to the midline and centered on the caudal level. Accurate positioning was achieved by regulating the micrometric screws and great care was taken to position the transducer in such a way as to avoid any compression of the skin (Figure 1D). In each mouse, an ultrasound cine-recording was acquired on the right and left sides of the back during cyclical passive trunk flexion using an articulated table with the hinge point of the table at the level of animal’s axilla. The table moved up and down at 0.5 Hz at a 17° angle (Figure 1E) from the horizontal for a total of four cycles.

### Ultrasound Image Measurement

**Figure 2:**
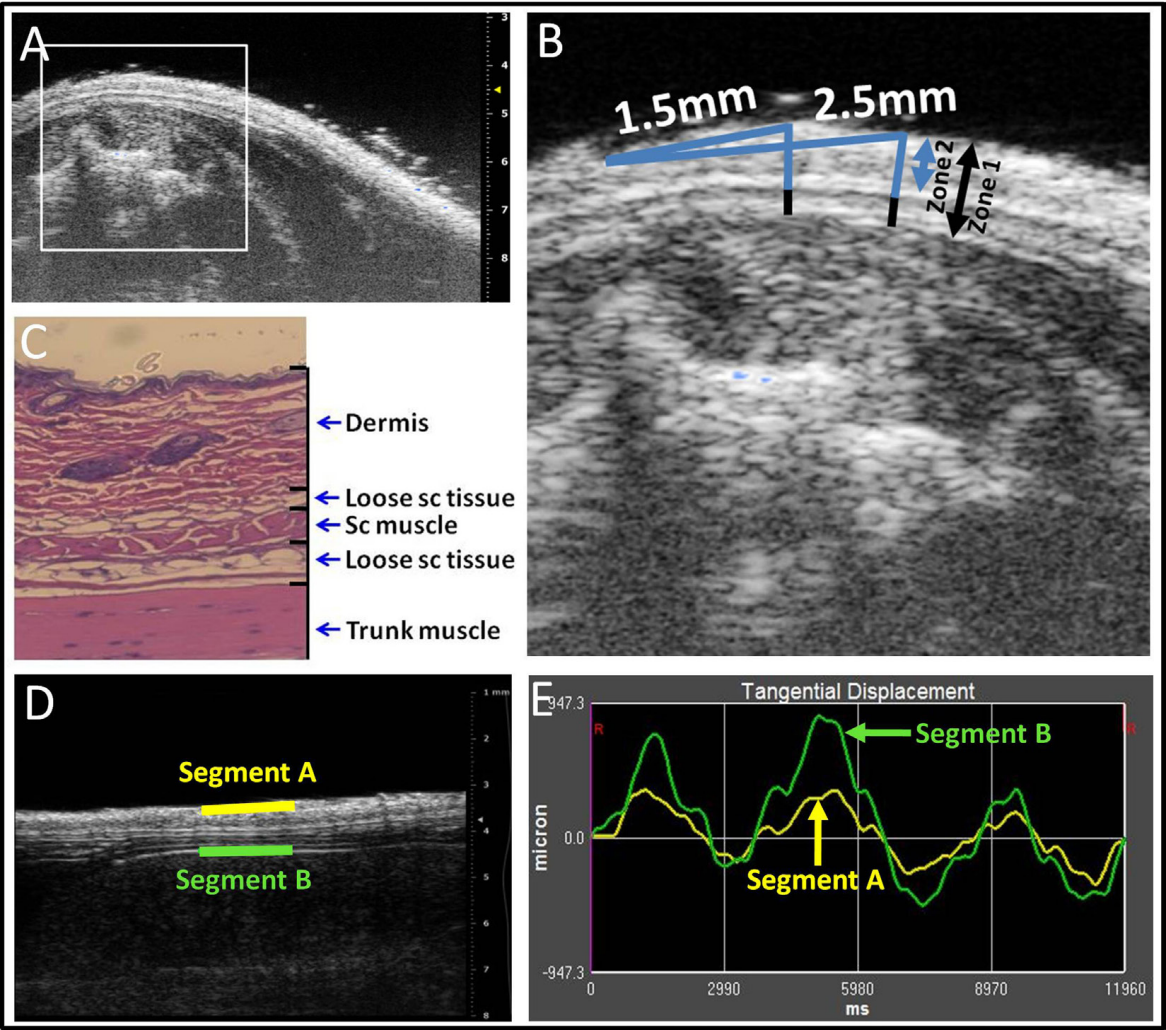
**Ultrasound image analysis methods**. **(A–C)**: ultrasound thickness measurements. Medial and lateral locations are, respectively, 1.5 and 2.5 mm from the midline **(A,B)**. Zones 1–3 are shown in ultrasound **(B)** and corresponding histology **(C)** image shows relation to dermis, loose subcutaneous (sc) tissue, subcutaneous (sc) muscle, and trunk muscle (erector spinae). **(D,E)**: Ultrasound measurement of tissue differential displacement. Segments a and b are, respectively, located on the superficial border of the dermis and the superficial border of the erector spinae perimuscular fascia. **(E)**: representative tracing of tangential displacement of superficial and deep segments shown in **(D)** (segment A yellow and segment B green).

For measurement of skin thickness, three zones were defined on the ultrasound images as illustrated in Figures 2A–C: Zone 1 (skin and subcutaneous tissue) extended from the superficial border of the dermis to the superficial border of the erector spinae muscle. Zone 2 (skin) extended from the superficial border of the dermis to the superficial border of the subcutaneous muscle, and Zone 3 from the superficial border of the subcutaneous muscle to the superficial border of the erector spinae muscle. Thickness measurements were performed at two fixed points, 1.5 mm (medial) and 2.5 mm (lateral) from the midline, respectively (Figure 2B). Thickness measurements at the four sites (upper and lower levels, medial, and lateral points) were averaged and taken as the outcome measure for Zones 1, 2, and 3 thickness.

For measurement of cumulative displacement, two segments were defined at the superficial border of the dermis (segment A) and the superficial border of the erector spinae perimuscular fascia (segment B), respectively (Figure 2D). Tissue displacement for each segment was measured using Vivovasc software as the tangential displacement during successive 40 ms increments over the two middle cycles of table motion (Figure 2E). Tangential displacement is defined as displacement along the direction of tissue planes (skin and perimuscular fascia). The absolute cumulative difference between the displacement of superficial and deep segments was calculated and taken as the outcome measure for relative tissue displacement. Ultrasound image measurement intra-rater reliability (ICCs) were *r* = 0.98 for tissue thickness and *r* = 0.87 for tissue displacement.

### Histopathological Assessment

At 4 weeks after splenocyte transfer, tissue specimens including skin, subcutaneous tissue, and muscle were excised from the shaved skin of one side of the back between the rib cage and the highest point of iliac crest, fixed in 10% neutral-buffered formalin, embedded in paraffin, and stained with standard hematoxylin and eosin (H&E). A blinded observer experienced in SSc pathology (Robert Lafyatis) scored H&E-stained back skin tissue samples per mouse for four parameters (fibrosis, inflammation, fat loss, and epidermal hypertrophy), using a semi-quantitative scale from 0 to 4. Values were summed to derive a combined histopathological score (13).

### Quantitative Real-time PCR

For mRNA expression studies, RNA was extracted from tissue samples corresponding to Zone 1 (skin and subcutaneous tissue) on the side contralateral to that used for histology. RNA was extracted using Trizol reagent (Qiagen, Santa Clarita, CA, USA) and reverse transcribed into cDNA with the Affinity Script CDNA Synthesis Kit (Agilent Technologies, Wilmington, DE, USA). Real-time quantitative PCR (qPCR) was performed using Sybr green reagent (Life Technologies, Grand Island, NY, USA). CT values for duplicate samples were averaged, and the amount of mRNA relative to a housekeeping gene transcript was calculated using the ΔCT method. Data were normalized to Hprt. The qPCR conditions were: 3 min 95°C, then 40 cycles of 10 s at 94°C, 10 s at 60°C, and 20 s at 72°C. Primers used for qPRC analysis are listed in Table 1.

**Table 1:** **Q-PCR primer sequence**.

Gene	Primer forward	Primer reverse
*Adam8*^a^	AGTTCCTGTTTATGCCCCAAAG	AAAGGTTGGCTTGACCTGCT
*Ccl2*^b^	GGCTCAGCCAGATGCAGTTAA	CCTACTCATTGGGATCATCTTGCT
*Hprt*^b^	GTTAAGCAGTACAGCCCCAAA	AGGGCATATCCAACAACAAACTT
*Il4ra*^b^	TCTGCATCCCGTTGTTTTGC	GCACCTGTGCATCCTGAATG
*Mmp12*^c^	GAACTTGCAGTCGGAGGGAA	TCTTGACAAGTACCATTCAGCA
*Tgfb1*^c^	CTTCAATACGTCAGACATTCGGG	GTAACGCCAGGAATTGTTGCTA
*Timp1*^a^	GCAACTCGGACCTGGTCATAA	CGGCCCGTGATGAGAAACT

*^a^Primers were selected from the Primer Bank website*.

*^b^Primers were described in Ref. (13)*.

*^c^Primers were designed with Primer Blast website tool*.

### *Ex Vivo* Tissue Explant Stretching Experiments and Fibroblast Morphological Measurements

Two separate *ex vivo* experiments were performed using subcutaneous connective tissue explants as previously described (24). In the first experiment, we compared four groups of mice without *in vivo* stretching (*N* = 4 mice per group): control mice euthanized at week 2, sclGvHD mice euthanized at week 2, control mice euthanized at week 4, and sclGvHD mice euthanized at week 4. The second experiment compared two groups: sclGVDH mice stretched *in vivo* for 4 weeks versus sclGVHD mice non-stretched (NS) *in vivo* for 4 weeks. In both experiments, immediately after euthanasia, a 8 cm × 3 cm tissue flap containing dermis, subcutaneous muscle, and subcutaneous tissue was excised from the back of the mouse. The tissue flap was cut into two pieces (right and left) that were randomized to *ex vivo* stretch versus control (no stretch). Each piece was placed transversely in grips and immersed in HEPES-physiological saline solution, pH 7.4 at 37°, containing (millimolars): NaCl 141.8, KCl 4.7, MgSO_4_ 1.7, EDTA 0.39, CaCl_2_ 2.8, HEPES 238.3, KH_2_PO_4_ 1.2, and Glucose 5.0. The grips and tissues were placed vertically in a tissue bath with the proximal grip connected to a 500 g (4.9 N) capacity load cell. Samples randomized to *ex vivo* stretch were elongated a rate of 1 mm/s by advancing a micrometer connected to the distal tissue grip to 25% strain relative to the unloaded length (length of the tissue laying flat but not stretched) then maintained at that length for the duration of the incubation. Control samples (randomized to *ex vivo* no stretch) were incubated for the same duration without stretch. At the end of incubation, the tissue was immersion-fixed in 4% paraformaldehyde for 1 h. After fixation, areolar subcutaneous connective tissue samples (each 10 mm × 10 mm) were dissected and placed flat on a glass slide and stained with Texas Red conjugated phalloidin (4 U/ml; Molecular Probes, Eugene, OR, USA) for 40 min at 4°C then counterstained for 2 min with DAPI (Molecular Probes, Eugene, OR, USA). Samples were mounted on slides using 50% glycerol in PBS with 1% *N*-propylgallate as a mounting medium and overlaid with a glass coverslip. Three slides were prepared from each sample, with an average of 3–4 images taken from each slide, avoiding the edges, using a 63× immersion oil lens and a digital camera AxioCam MRC 5, Zeiss. Images were imported into the analysis software package Image J for morphometric analysis. An average of 30 fibroblasts were measured per excised tissue sample by a blinded investigator. A cell was excluded if part of its cell body perimeter is outside the image. For each cell, the cell body perimeter was traced as defined as the outline of the cell’s cytoplasm projected in the plane of the image excluding cell processes (defined as an extension of a cell’s cytoplasm longer than 2 μm and less than 2 μm in width at any portion of its length). Fibroblast cell body cross-sectional area was calculated as the area delimited by the cell body perimeter projected onto the image plane (see Figure 8 for illustration of method).

### Statistical Analysis

Repeated measures analyses of variance were performed to examine the effects of group, time, and their interaction. Dependent measures were zone-specific tissue thickness, relative tissue displacement, clinical score, and weights. Because group differences were found to be time-dependent (i.e., group × time interaction *p* < 0.05 for all outcome measures), partial *F*-tests were used to compare the four groups at each time point. If the partial *F*-test was significant, Fisher’s Protected LSD was used to perform pairwise comparisons among groups at each time point to control type I error rate experiment-wise. For analyses of clinical score, control animals were excluded as all values were scored as 0. A one-way ANOVA was used to compare the four groups on histopathological score and mRNA expression. For mRNA, analyses of variance were performed on log fold change values, with results presented as geometric means and associated SEs. In a subset of animals (*N* = 5), estimates of intra-rater reliability were computed using on intraclass correlation coefficients for both tissue thickness and displacement. These estimates were based on variance components that were derived based on mixed model analyses of variance. Correlation between outcome variables were examined using Spearman’s correlation. Repeated measures ANOVAs were used to examine *in vivo* and *ex vivo* stretch effects on fibroblast cross-sectional area. All statistical analyses were performed using SAS statistical software Version 9.4 (SAS Institute, Cary, NC, USA). Statistical significance was determined based on α = 0.05.

## Results

### Clinical Score, Animal Weight, and Histological Score

**Figure 3:**
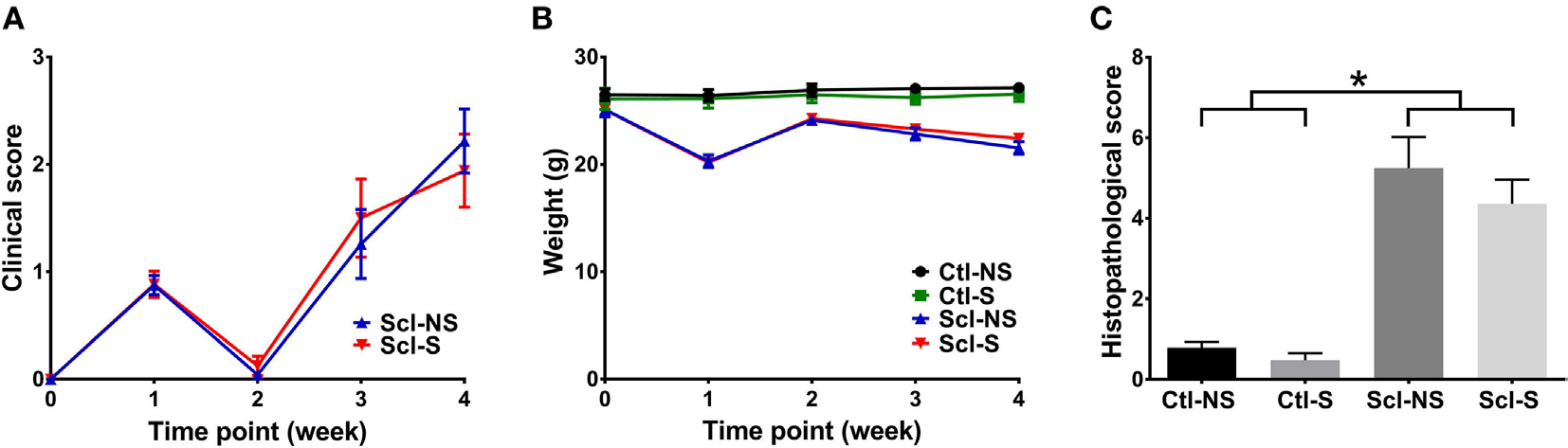
**Clinical and histological measures**. **(A,B)**: Clinical score **(A)** and animal weight **(B)** over the course of the 4-week experiment. **(C)**: Histological scores at week 4. Significant differences are shown between both sclGvHD/stretch (Scl-S) and sclGvHD/no stretch (Scl-NS) compared to both Con-S and Con-NS groups at week 2 (*p < 0.05). There were no significant differences between Scl-S and Scl-NS groups in any of the outcome measures (*N* = 12 mice per group).

Clinical scores initially increased from baseline to week 1, subsequently decreased from week 1 to week 2 and rose again at weeks 3 and 4 (Figure 3A). This is consistent with previous studies that identified successive phases of skin involvement in the sclGvHD model, with an early predominantly inflammatory phase followed by fibrosis and atrophy (15–17). sclGvHD animal weights paralleled the clinical scores by first decreasing at week 1, rebounding at week 2 and decreasing again at weeks 3 and 4 compared with controls (Figure 3B). Mean combined histological scores at week 4 for the Scl-NS group was 5.3 ± 0.5 which is consistent with previous reports using this model (25) (Figure 3C). Animal weights were lower in both Scl groups compared with both control groups at weeks 1–4. Differences in clinical scores, weight, and histological scores between Scl-NS and Scl-S groups were not statistically significant (Figures 3A–C; Table 2).

**Table 2:** **Repeated measurements at weekly time points**.

	Time point (week)	Control/no stretch *N* = 12	Control/stretch *N* = 12	sclGvHD/no stretch *N* = 12	sclGvHD/stretch *N* = 12	*p*-Value (ANOVA)
Thickness Zone 1 (μm)	1	489.14 ± 13.97^a^	488.06 ± 17.77^a^	472.77 ± 16.30^ab^	462.60 ± 11.35^b^	0.040
	2	486.50 ± 9.82^a^	485.15 ± 14.84^a^	606.08 ± 14.22^b^	599.06 ± 15.76^b^	<0.001
	3	491.67 ± 9.27^a^	479.52 ± 16.30^a^	554.85 ± 16.26^b^	503.19 ± 13.50^a^	<0.001
	4	473.29 ± 17.92	476.96 ± 18.47^a^	463.04 ± 13.93^ab^	448.40 ± 16.94^b^	0.037
Thickness Zone 2 (μm)	1	327.29 ± 11.39^a^	345.52 ± 16.10^a^	327.54 ± 16.64^a^	320.46 ± 9.75^a^	0.060
	2	319.10 ± 9.27^a^	320.38 ± 12.27^a^	410.23 ± 11.06^b^	410.75 ± 16.69^b^	<0.001
	3	319.25 ± 12.75^a^	324.75 ± 14.43^a^	395.77 ± 15.91^b^	355.75 ± 13.05^a^	<0.001
	4	312.06 ± 17.48^a^	326.40 ± 17.42^a^	313.35 ± 12.91^a^	309.46 ± 15.46^a^	0.120
Thickness Zone 3 (μm)	1	161.17 ± 6.37^a^	142.54 ± 5.50^b^	145.23 ± 2.71^b^	142.15 ± 4.79^b^	0.040
	2	167.40 ± 4.46^a^	164.77 ± 4.20^a^	195.85 ± 8.38^b^	188.31 ± 6.27^b^	<0.001
	3	172.42 ± 7.61^a^	154.77 ± 3.60^b^	159.08 ± 4.98^ab^	147.44 ± 4.44^b^	0.011
	4	161.23 ± 3.70^a^	150.56 ± 3.78^ab^	149.69 ± 5.77^ab^	138.94 ± 5.50^b^	0.037
Cumulative displacement (μm)	1	429.98 ± 44.92^a^	432.64 ± 28.59^a^	381.75 ± 34.95^a^	364.27 ± 42.33^a^	0.238
	2	422.20 ± 52.54^a^	464.33 ± 29.66^a^	416.84 ± 37.61^a^	455.58 ± 49.48^a^	0.629
	3	456.93 ± 35.99^a^	444.10 ± 33.98^a^	344.88 ± 31.90^b^	430.00 ± 30.34^a^	0.037
	4	457.39 ± 33.13^ab^	473.83 ± 23.88^a^	331.28 ± 38.32^c^	388.45 ± 22.28^bc^	0.003
Clinical score	1	N/A	N/A	0.88 ± 0.09^b^	0.88 ± 0.12^b^	0.961
	2	N/A	N/A	0.04 ± 0.04^b^	0.13 ± 0.09^b^	0.864
	3	N/A	N/A	1.26 ± 0.32^b^	1.50 ± 0.36^b^	0.524
	4	N/A	N/A	2.22 ± 0.30^b^	1.94 ± 0.34^b^	0.378
Weight (g)	1	26.40 ± 0.55^a^	26.10 ± 0.86^a^	20.30 ± 0.58^b^	20.17 ± 0.40^b^	<0.001
	2	26.90 ± 0.58^a^	26.46 ± 0.73^a^	24.13 ± 0.44^b^	24.26 ± 0.38^b^	0.001
	3	27.06 ± 0.48^a^	26.22 ± 0.63^a^	22.83 ± 0.57^b^	23.30 ± 0.45^b^	<0.001
	4	27.13 ± 0.51^a^	26.53 ± 0.70^a^	21.53 ± 0.59^b^	22.41 ± 0.48^b^	<0.001

*Results are expressed as mean ± SE. Means for experimental conditions within each time point*.

*Not sharing a common letter are significantly different (*p* < 0.05, Fisher’s protected LSD)*.

### Ultrasound Measurement of Tissue Thickness

**Figure 4:**
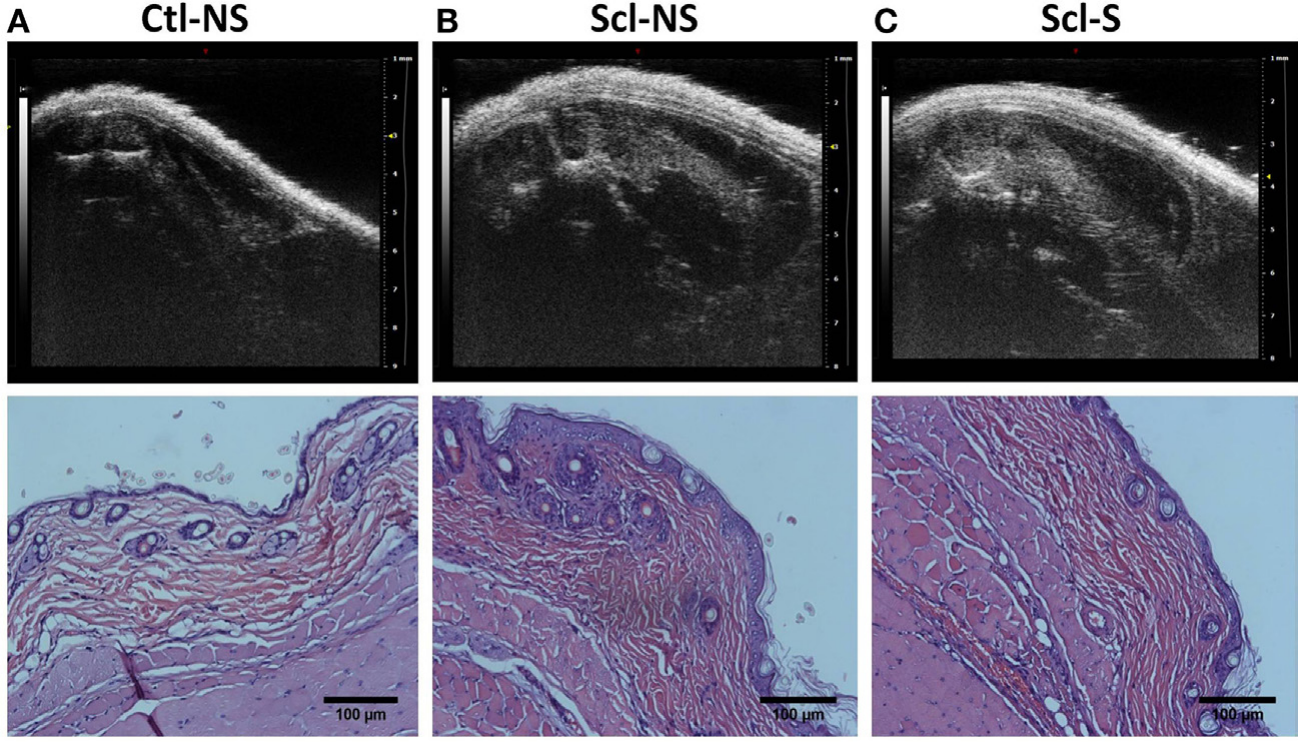
**Ultrasound and histology images at week 4**. Ultrasound and corresponding histology images were taken from the same location on the back (at the location indicated in Figure 2A). **(A)** Control no stretch; **(B)** sclerodermatous graft-versus-host disease (SclGvHD) no stretch; **(C)** sclGvHD-stretch. Histological images show increased dermal collagen density in both non-stretched and stretched sclGvHD mice.

**Figure 5:**
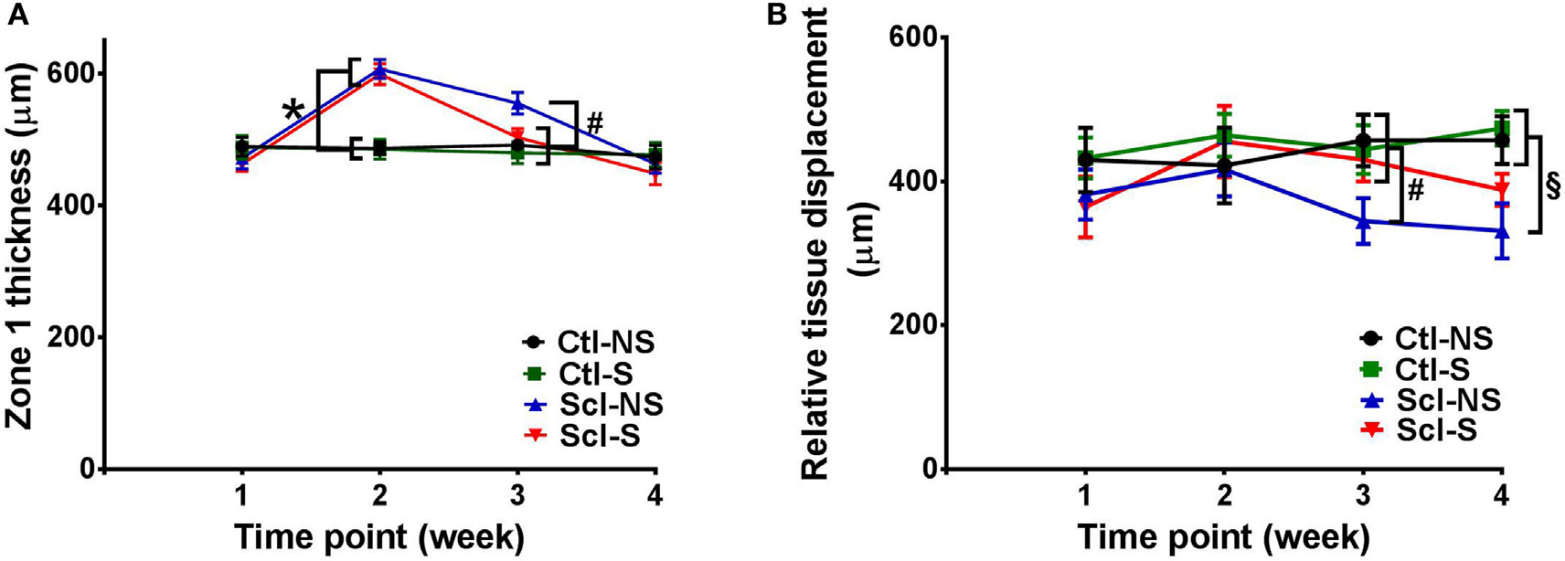
**Ultrasound measurement of tissue thickness (A) and relative tissue displacement (B) over the course of the 4-week experiment in the four experimental groups**. **(A)** Significant differences (*p* < 0.05) are shown between both sclGvHD/stretch (Scl-S) and sclGvHD/no stretch (Scl-NS) compared to both Ctl-S and Ctl-NS groups at week 2 (*) and between Scl-NS and the other three groups at week 3 (#). **(B)** Significant differences are shown between Scl-NS and the other three groups at week 3 (#) and between the Scl-NS and both control groups at week 4 (¶). Scl-S and Scl-NS were not significantly different at week 4 (*N* = 12 mice per group).

Figure 4 shows examples of ultrasound and corresponding histology images for control (Figure 4A) and sclGvHD (Figures 4B,C) mice illustrating the greater dermal thickness in the sclGvHD mice visible with both ultrasound and histology. Ultrasound measurements of Zone 1 (combined skin and subcutaneous tissue), Zone 2 (skin only), and Zone 3 (subcutaneous tissue only), all demonstrated time-dependent differences between the four experimental groups (ANOVA time by group interaction *p* = 0.001) (Figure 5A; Table 2). Skin and subcutaneous tissue thickness peaked at week 2 in the sclGvHD mice, which is consistent with the presence of early inflammatory edema.

Stretching had a significant effect on Zone 1 (combined skin and subcutaneous tissue) and Zone 2 (skin only) thickness, which were both reduced by 11% in the Scl-S group compared with the Scl-NS group at week 3 (Figure 5A; Table 2). Thus, while there was no difference between stretch and no stretch at the peak of inflammation (week 2), stretched sclGvHD mice became more similar to controls at week 3.

### Ultrasound Measurement of Tissue Mobility

Ultrasound measurement of relative tissue displacement between skin and subcutaneous tissue during passive trunk flexion also showed significant differences between groups (ANOVA *p* = 0.01) (Figure 5B; Table 2). Although the interaction between group and time was not significant, differences between groups were significant at weeks 3 and 4. At week 3, relative tissue displacement was 26% greater in the Scl-S group compared with the Scl-NS group (Figure 5B; Table 2). At week 4, relative tissue displacement remained greater in the Scl-S compared with Scl-NS, but this difference was no longer statistically significant (Figure 5B; Table 2). There were no significant differences between experimental groups in the simple displacements of either skin or subcutaneous tissue during the passive trunk motion, indicating that stretching had a specific impact on the relative inter-layer mobility of tissues rather than the absolute amount of tissue displacement. Thus, as in the thickness measurements, stretching improved tissue mobility such that stretched sclGvHD mice were more similar to controls at week 3 only.

### Gene Expression Analysis

**Figure 6:**
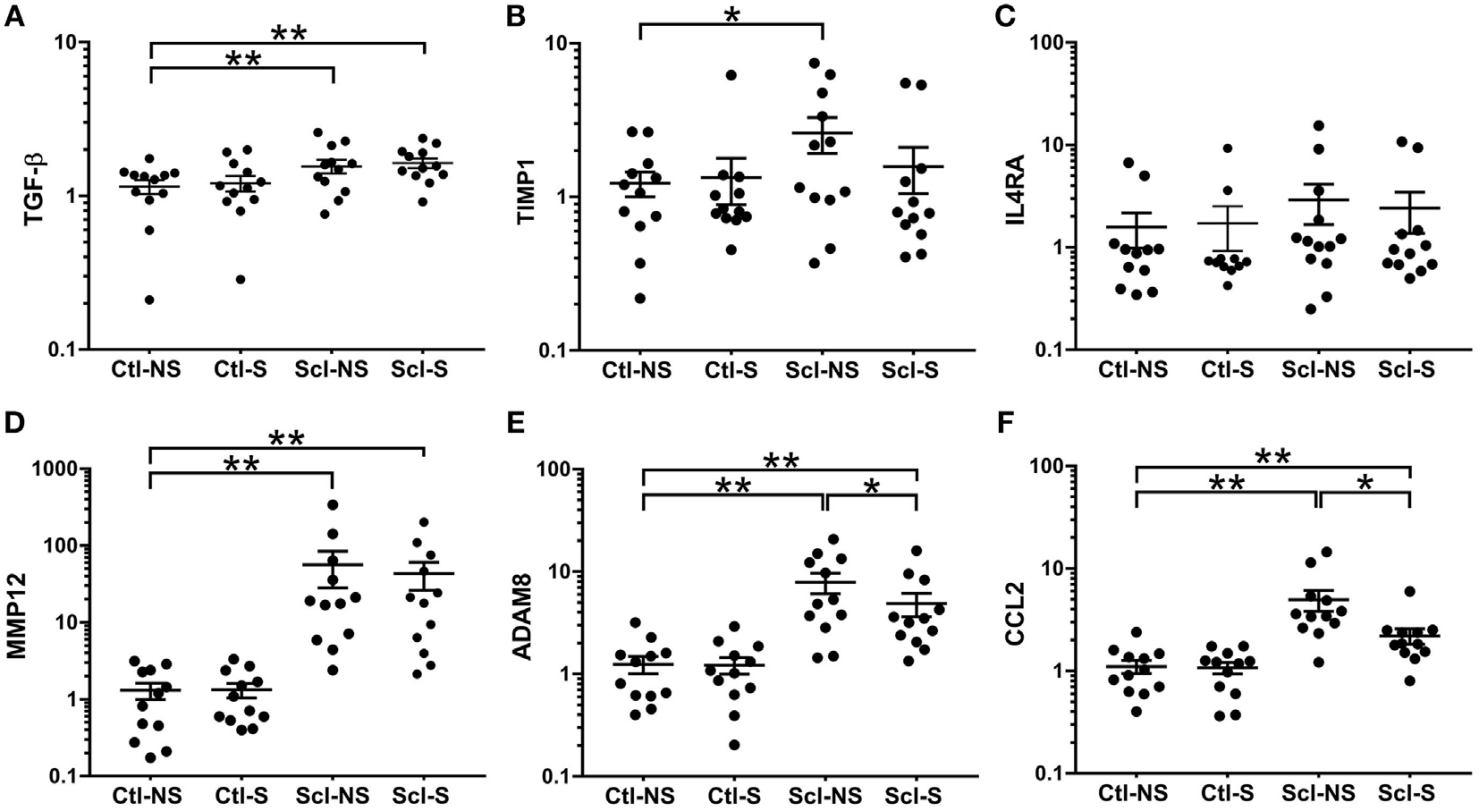
**mRNA expression (qPCR) analyses**. **(A–F)**: Significance levels for sclGvHD/no stretch and sclGvHD/stretch groups are shown relative to control/no stretch (Ctl-NS) group (*p < 0.05, **p < 0.01, *N* = 12 mice per group). There were no significant differences between Ctl-NS and Ctl/stretch groups for any of the outcome measures. Results are presented as geometric means and associated SEs, with the *y*-axis reflecting log scale.

At euthanasia (week 4), we measured markers of fibrosis (TGF-β, TIMP1, MMP12, ADAM8) and inflammation (IL4RA, CCL2) that have been previously shown to be upregulated in both sclGvHD and SSc patients. RNA expression was significantly greater in the Scl-NS compared to the Ctl-NS group for TGF-β *p* = 0.006, TIMP1 *p* = 0.012, MMP12 *p* < 0.001, ADAM8 *p* < 0.001, and CCL2 *p* < 0.001 (Figure 6). Stretching significantly reduced the expression of ADAM8 and CCL2 compared with no stretching in sclGvHD mice (*p* = 0.011 and *p* < 0.001, respectively) (Figures 6E,F).

In order to further investigate possible anti-inflammatory effects of stretching suggested by the reduction in CCL2 at week 4, we conducted additional experiments in which mice randomined to *in vivo* stretch versus no stretch were euthanized at the peak of inflammation (week 2) for measurement of CCL2 along with additional markers of inflammation and matrix remodeling (COX2, PAK2, PLA2, YAP, CTGF, Adam8, MMP12, TGF-β, IL13, IL13ra1, IL4ra, and TIMP1). Although all markers were elevated in the SclGVHD mice compared with controls, we found no significant differences at week 2 between stretched and NS mice for any of the markers, including CCL2 (data not shown). This suggests that the reduced skin thickness and increased mobility observed at week 3 was not due to an early interruption of the inflammatory cascade.

### Correlation Analysis

**Figure 7:**
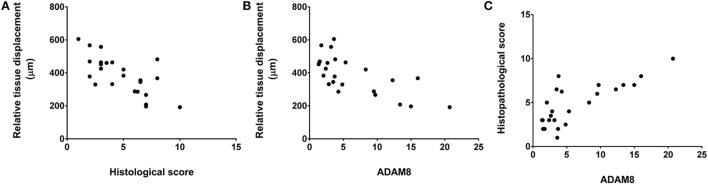
**Relationship of relative tissue displacement, histological score, and ADAM8 mRNA expression**. Spearman’s correlation coefficients were *r* = −0.70, *p* ≤ 0.001 **(A)**, *r* = −0.69, *p* < 0.001 **(B)**, and *r* = 0.77, *p* < 0.001 **(C)**.

We did not observe any significant correlation between tissue thickness and either histological score, clinical score, or gene expression measures. However, relative tissue displacement was negatively correlated with both histopathological score (*r* = −0.70, *p* = < 0.001) and ADAM 8 expression in tissues of the back (*r* = −0.69, *p* < 0.001), and ADAM 8 was positively correlated with the histopathological score (*r* = 0.77, *p* < 0.001) (Figure 7).

### *Ex Vivo* Stretching Experiments

**Figure 8:**
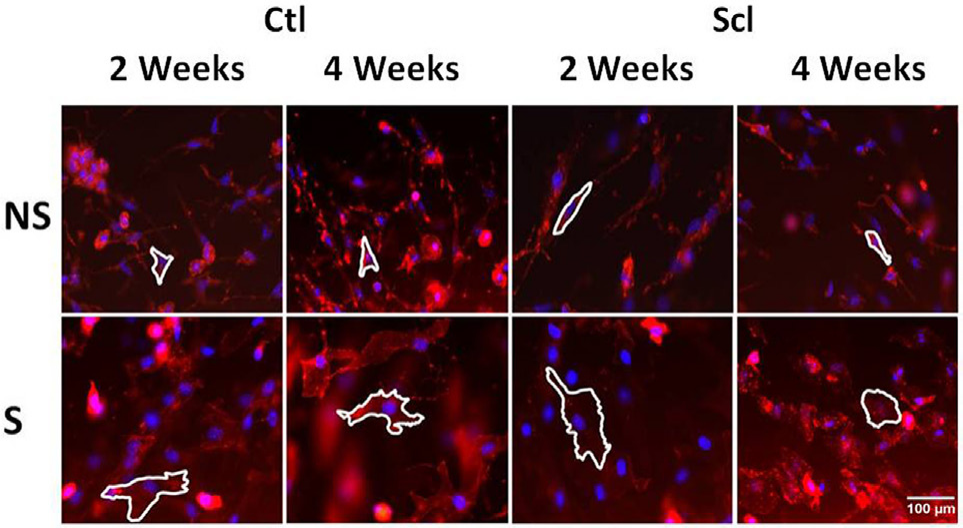
**Fibroblast cytoskeletal response to tissue stretch *ex vivo***. Tissue explants from Control and sclerodermatous graft-versus-host disease mice were excised immediately after euthanasia at week 2 or week 4, then stretched (S) versus non-stretched *ex vivo* for 2 h, followed by fixation and staining with phalloidin for measurement of cell body cross-sectional area (outlined as described in Section “Materials and Methods”).

**Figure 9:**
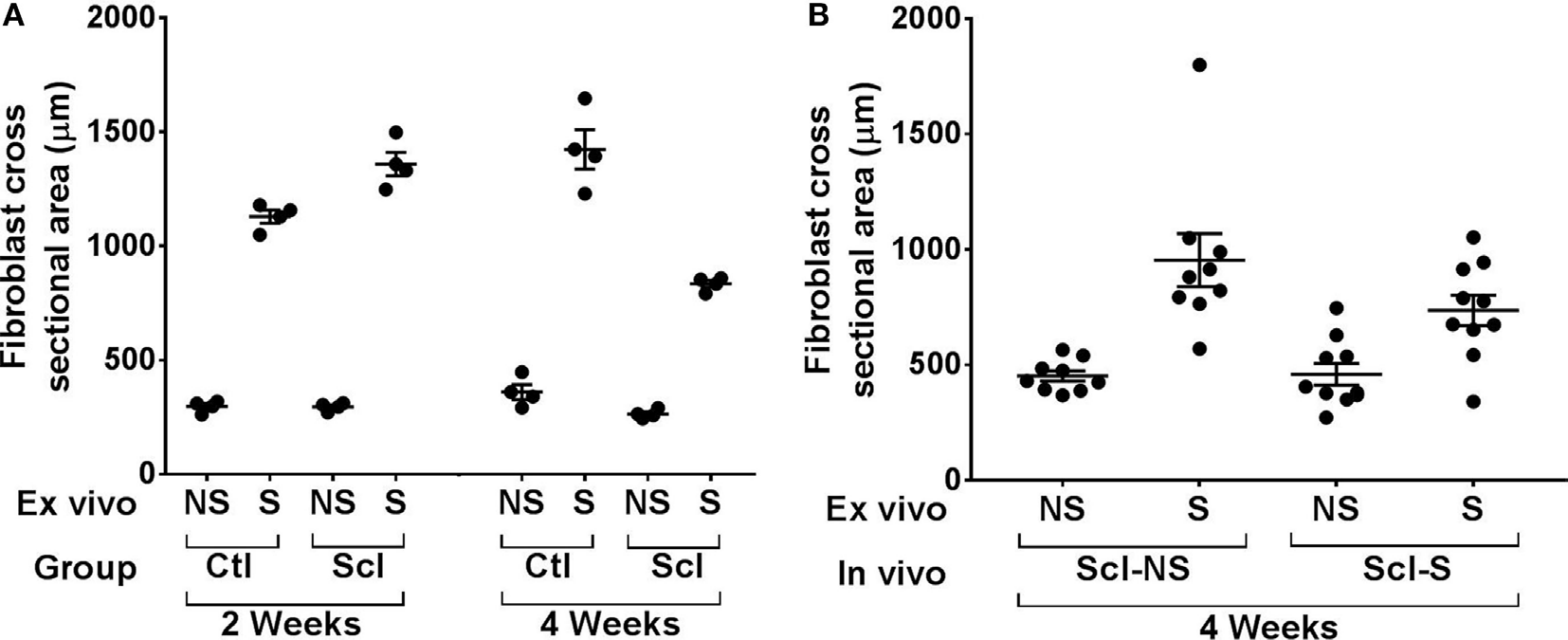
***Ex vivo* stretching experiments**. **(A)**: Connective tissue explants from control and sclerodermatous graft-versus-host disease (sclGvHD) mice were stretched (S) versus not stretched for 2 h *ex vivo*, then fixed and stained for measurement of fibroblast cross-sectional area as shown in Figure 8. Fibroblast response to *ex vivo* tissue stretch was reduced in sclGvHD mice that were euthanized at week 4 but not week 2, compared with controls. **(B)**: In sclGvHD mice, daily *in vivo* stretching did not result in an increased fibroblast responsiveness to *ex vivo* stretching at week 4.

In order to investigate the possible role of connective tissue fibroblasts in the response to stretching in the sclGvHD model, we conducted experiments in which connective tissue samples from control and sclGvHD mice are stretched *ex vivo*. We previously showed that connective tissue fibroblasts respond to tissue stretch *ex vivo* with cytoskeletal expansion accompanied by a drop in tissue tension (26) and that this fibroblast response is impaired in cross-linked collagen gels (27). We hypothesized that loss of fibroblast responsiveness to stretching may contribute to increased tissue stiffness in sclGvHd, and that stretching *in vivo* may help preserve fibroblast responsiveness as well as tissue mobility. We first tested whether fibroblast responsiveness to stretching *ex vivo* is impaired in sclGvHD compared with controls. We measured fibroblast cross sectional area in tissue explants stretched versus non-stretched *ex vivo* from four groups of mice: control week 2, sclGvHD week 2, control week 4, and sclGvHD week 4 (Figure 8). We found that, at week 2, fibroblasts in explants from control and Scl GvHD responded similarly to *ex vivo* stretching (main effect of *ex vivo* stretch *p* < 0.001). However, at week 4, fibroblast expansion was reduced in explants from sclGvHD mice compared with controls (group by stretch interaction *p* < 0.01) (Figure 9A). We then tested whether *in vivo* stretching for 10 min/day *in vivo* would prevent the loss of fibroblast responsiveness *ex vivo* at week 4 and found no significant difference between mice that were stretched versus non-stretched *in vivo* (Figure 9B). Thus, while cytoskeletal expansion and remodeling of connective tissue fibroblasts is clearly impaired in sclGvHD, this response was not improved by *in vivo* stretching. This suggests that fibroblast-mediated tissue relaxation is not part of the mechanism underlying the beneficial effect of stretching.

## Discussion

In this study, we show that *in vivo* ultrasound is a valuable technique to monitor skin thickness and subcutaneous tissue mobility in the murine sclGvHD model. In addition, daily stretching produced a measurable beneficial effect since it reduced skin thickness and improved mobility during the fibrotic phase of the model (week 3). However, we found no differences in weight loss, clinical and histological scores between stretch and no stretch groups. A possible interpretation for these results is that our ultrasound findings were unrelated to any meaningful clinical improvement. On the other hand, it is also possible that ultrasound measurements are more sensitive than clinical and histological scores in following the course of the disease, since a marked increase in skin thickness in the sclGvHD mice was detectable with ultrasound at week 2 in the absence of clinical symptoms. This may be because the clinical score focuses on alopecia (and marginally periorbital swelling) which is more externally visible than skin swelling. Furthermore, clinicial scores do not reflect lack of tissue mobility which is one of the main sources of impairment in SSc. Even though clinical and histological scores were not different between stretch and no stretch groups, the correlation between skin mobility and histological scores suggests that skin mobility measured with ultrasound may be a useful and sensitive non-invasive marker in this model.

The mechanisms underlying any beneficial effect of stretching on skin thickness and mobility are at present unclear. Stretching reduced mRNA expression of CCL2 which is an important inflammatory mediator associated with the sclGvHD model (13, 14). The lack of accompanying reduction in clinical symptoms stands in contrast to a previous study using this model in which treatment with CCL2 antibodies completely prevented the development of clinical and tissue pathology (13). However, CCL2 antibodies were administered systemically beginning immediately after cell transfer while, in our study, we only saw stretch-induced reduction of CCL2 at week 4 and not at the peak of inflammation (week 2). It is, therefore, possible that reduction in CCL2 by stretching was not sufficient in magnitude or occurred too late to have a significant impact on the inflammatory process. Furthermore, we did not observe any reduction of inflammatory genes at week 2, suggesting that the reduced skin thickness and increased mobility observed at week 3 was not due to an early interruption of the inflammatory cascade.

Another possibility is that the effect of stretching is not primarily anti-inflammatory, but rather is mechanical in nature. Reduced shear plane mobility of subcutaneous connective tissue can result from a number of interrelated factors, including adhesions between tissue layers and increased connective tissue matrix stiffness due to increased collagen deposition and cross linking. We found no significant reduction in TGF-β, TIMP-1, or MMP-12 with stretching, suggesting that stretching did not directly impact fibrotic matrix remodeling. We also found no improvement of fibroblast responsiveness to tissue stretch *ex vivo* in sclGvHD mice that had been stretched *in vivo*, suggesting that tissue stiffness remained higher than in controls in both stretched and NS sclGvHD mice at week 4. This is consistent with our ultrasound findings showing further reduction in tissue mobility from week 3 to week 4 in both sclGvHD groups compared with controls. On the other hand, although *in vivo* stretching did not fully reverse the pathology, the reduction of ADAM8 mRNA expression in stretched mice and positive correlation of ADAM8, tissue mobility, and histological scores suggests a role for cell–matrix interactions in the effect of stretching, since matricellular proteins of the ADAM family have been implicated in mechanical signaling as well as inflammation (28, 29). Further studies will be needed to specifically examine the contribution of extracellular matrix stiffness and cell–matrix interactions to the effects of *in vivo* stretching.

Although mechanisms are at this point unclear, it is important to note that the observed effects of stretching occurred in the absence of any other treatment. The implication of these pre-clinical findings is that a systematic investigation of stretching as a therapeutic modality is warranted in patients with SSc. This is particularly important in light of a recent report that only 10% of SSc patients received physical/occupational therapy and that a frequent complaint from patients is that physical therapists are often reluctant to treat patients with SSc (6). The magnitude of improvement in tissue mobility (25%) observed in our study was comparable to improvements in range of motion of joints and other tissues that are considered clinically significant in other conditions (30), as well as the magnitude of clinical improvement typically found with commonly prescribed pharmacological treatments in patients with diffuse sclerodermatous skin involvement (31, 32). Although the presence of deep tissue “friction rubs” has been reported as a clinical sign associated with poor prognosis and was suggested as a routine part of the rheumatologic physical examination (33), there are currently no established methods to quantify the involvement of deep tissues. Dynamic ultrasound imaging has been successfully used in humans to measure the mobility of connective tissues (19), but so far this technique has not been applied to scleroderma. Thus, a next step in the translation of our current results to humans would be the development and validation of a clinical protocol using dynamic ultrasound to first follow the course of the disease, and then quantify the effects of treatments, including stretching.

## Author Contributions

All the authors reviewed the final version and are accountable for the accuracy and integrity of results. YX contributed to data acquisition, analysis, and interpretation and drafted the manuscript. HL, AA, and LB designed the study, contributed to data analysis and interpretation, and revised the manuscript. KU, SO, IM, and RL contributed to data acquisition and revised the manuscript. GB contributed to the study design, data analysis and interpretation, and revised the manuscript.

## Conflict of Interest Statement

The authors declare that the research was conducted in the absence of any commercial or financial relationships that could be construed as a potential conflict of interest.
